# Pilot data of serum proteins from children with autism spectrum disorders

**DOI:** 10.1016/j.dib.2019.104558

**Published:** 2019-09-25

**Authors:** Anna L. Kaysheva, Alexander A. Stepanov, Artur T. Kopylov, Tatiana V. Butkova, Tatyana Pleshakova, Vasily V. Ryabtsev, Ivan Yu. Iourov, Svetlana G. Vorsanova, Yuri D. Ivanov

**Affiliations:** a Institute of Biomedical Chemistry^1^, Russia; b Mental Health Research Centre, Russia; c Veltischev Research and Clinical Institute for Pediatrics of the Pirogov Russian National Research Medical University, Moscow, Russia

**Keywords:** Autism spectrum disorder, Panoramic mass spectrometry, Label-free quantitative analysis

## Abstract

Protein profiles of 13 serum samples from children with autism spectrum disorders (ASD) and 11 serum samples from healthy volunteers was obtained using panoramic ultra-high resolution mass spectrometry. The analysis of measurements was performed using the proteomics search engine. We identified a group of 74 proteins which we term a “protein fingerprint” specific for serum samples collected from children with autism. Components of the protein fingerprint are involved in hemostasis maintenance including biological regulation, the response to stimulus, regulation of metabolism, and proteins of the immune system.

**Table:** Specifications Table

Subject area	Biology
More specific subject area	Biochemistry, Proteomics, Label-free Quantitative Analysis of Protein
Type of data	Tables, figures
How data was acquired	Liquid chromatography-tandem mass spectrometric analysis was carried out using Q Exactive high resolution mass spectrometer (Thermo Scientific, USA) by chromatographic separation using Ultimate 3000 Nano-flow HPLC system (Thermo Scientific, USA)
Data format	Raw, filtered, analyzed
Experimental factors	The trypsin digestion was used for depleted serum samples
Experimental features	13 depleted serum samples were drawn from children with ASD and 13 ones from healthy volunteers. Minor proteins were supplemented using a column MARS Hu-14. Enzymatic cleavage of proteins was performed using trypsin. HPLC-MS/MS registration of peptides was carried out using Q Exactive mass spectrometer (Thermo Scientific, USA) by chromatographic separation using Ultimate 3000 Nano-flow HPLC system (Thermo Scientific, USA).
Data source location	Moscow, Russia
Data accessibility	The mass spectrometry proteomics data have been deposited to the Proteome XchangeConsortium (http://www.proteomexchange.org/) via the PRIDE partner repository with the dataset identifier PXD005193. Other datasets are directly provided with this article.
Related research article	Kaysheva AL, Kopylov AT, Pleshakova TO, Iourov IY et al. Proteomic analysis of serum proteins of children with autism. Biotecnologia Aplicada. 2017, 34(1), 2211–2214.

**Table:** 

**Value of the Data** • The data including the raw data of protein and peptide identification and quantization can be used by other scientists investigating molecular basis of autism spectrum disorders. • The data provide a comparative analysis of protein profiles from samples derived from children with ASD. It was found that the protein profile of patients with ASD differs from that of healthy volunteers. • Protein abundances, presence and variance in the samples after depletion are of potential value to determine which bioinformatic method can be useful for proteomics investigations. • The bioinformatics data can provides insight into the biological function of the successfully identified proteins.

## 1 Data

**Fig. 1:**
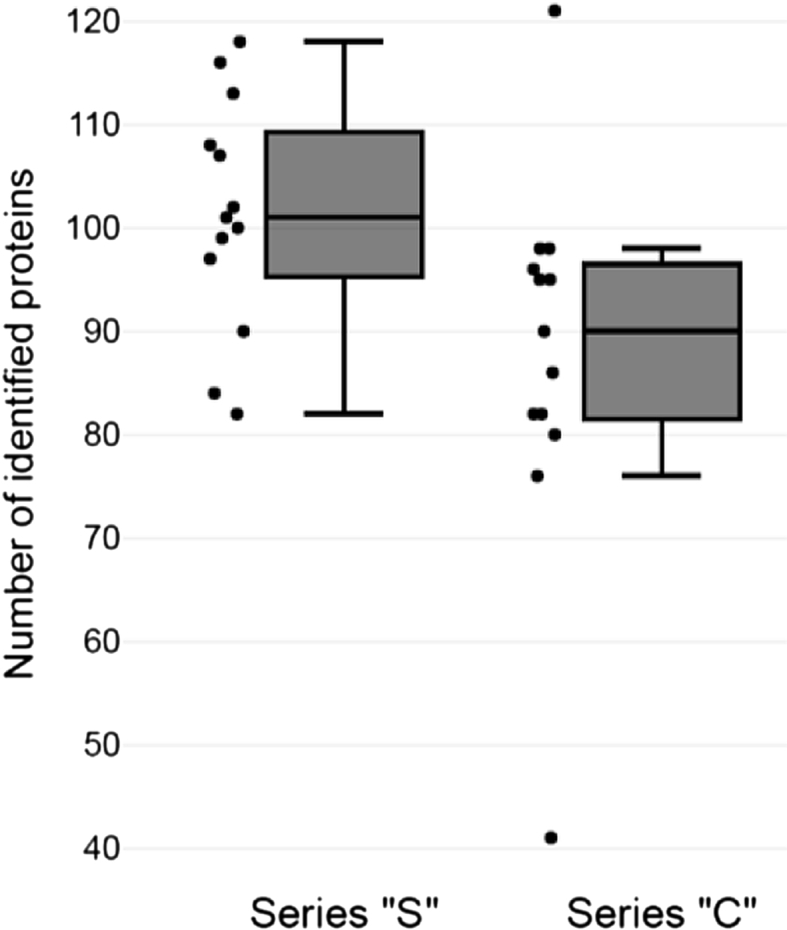
Number of identified proteins in depleted serum samples. Box plots of total number of identified proteins in control serum samples (“С” series) and serum samples from patients with ASD (“S” series). These box plots show a box bounded by the interquartile range (IQR; 25th to 75th percentile), with the median (50th percentile) inside box; whiskers extending either or 1.5 IQR above or below the box; outliers beyond the whiskers (solid dots).

**Fig. 2:**
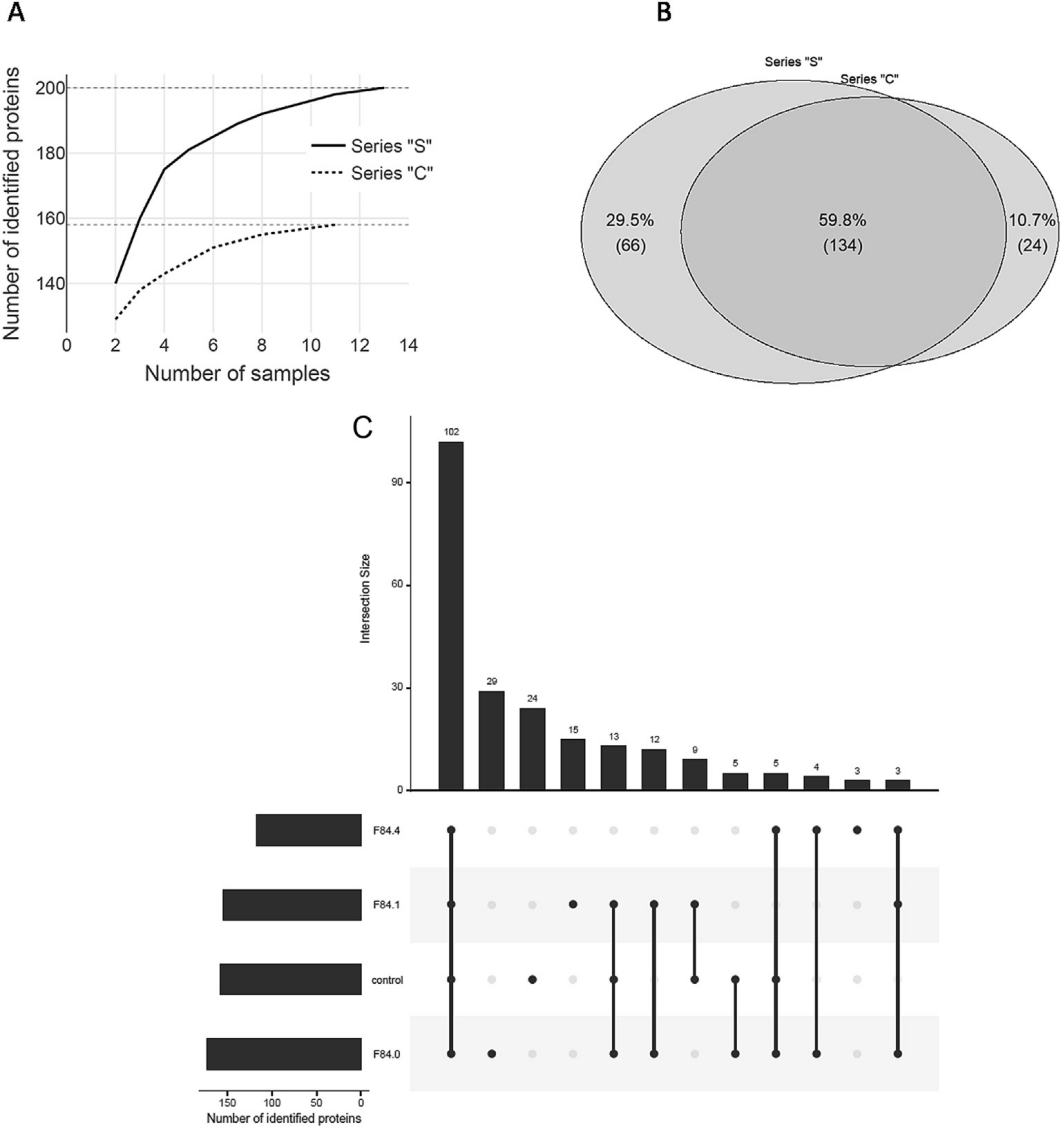
Saturation curve of identified proteins depending on the number of analyzed samples in series “S” (solid line) and series “С” (dotted line) (А). Matches in protein identifications between depleted control samples (“С” series) and serum samples from children with ASD (“S” series) (В). The UpSet diagram shows the intersection size among ASD diagnosis and series “Control”.

MS revelation of protein composition of 13 depleted serum samples drawn from children with ASD and 13 ones from healthy volunteers indicate that the samples are quite similar in both the number of identified proteins (Fig. 1), and in protein composition (Fig. 2B). We observed that the median for the number of identifications in samples of “S” series is 10% higher than that of the control series and amounts to 100 proteins. The median for the number of identifications in the samples of “C” series is 90 proteins. In “C” series we found two samples of Outliers (Fig. 1, marked with green dots), the number of identified proteins for which falls outside 1,5 IQR.

We formed two protein comparison lists: (1) the control list contains proteins (n = 158) that had been detected in series “C”, (2) the list of proteins (n = 200) associated with ASD contains proteins that had been detected in series “S”. 134 proteins occurred in both series (Fig. 2B).

In order to determine whether samples are qualitatively representative of total composition of proteins for each series, we built up the cumulative relationship between the number of identified proteins and sample range (Fig. 2A). Each protein discovery (accumulation) curve is a graphic expression of the cumulative number of identified proteins as a function of the cumulative number of persons.

Hence, curves in Fig. 2A characterize samples with regard to the diversity of protein identifications and the sufficient number of samples required to attain the maximum number of identifications (plateau) in the series. The plateau height corresponds to the size of ellipse in the Venn diagram (Fig. 2B).

**Fig. 3:**
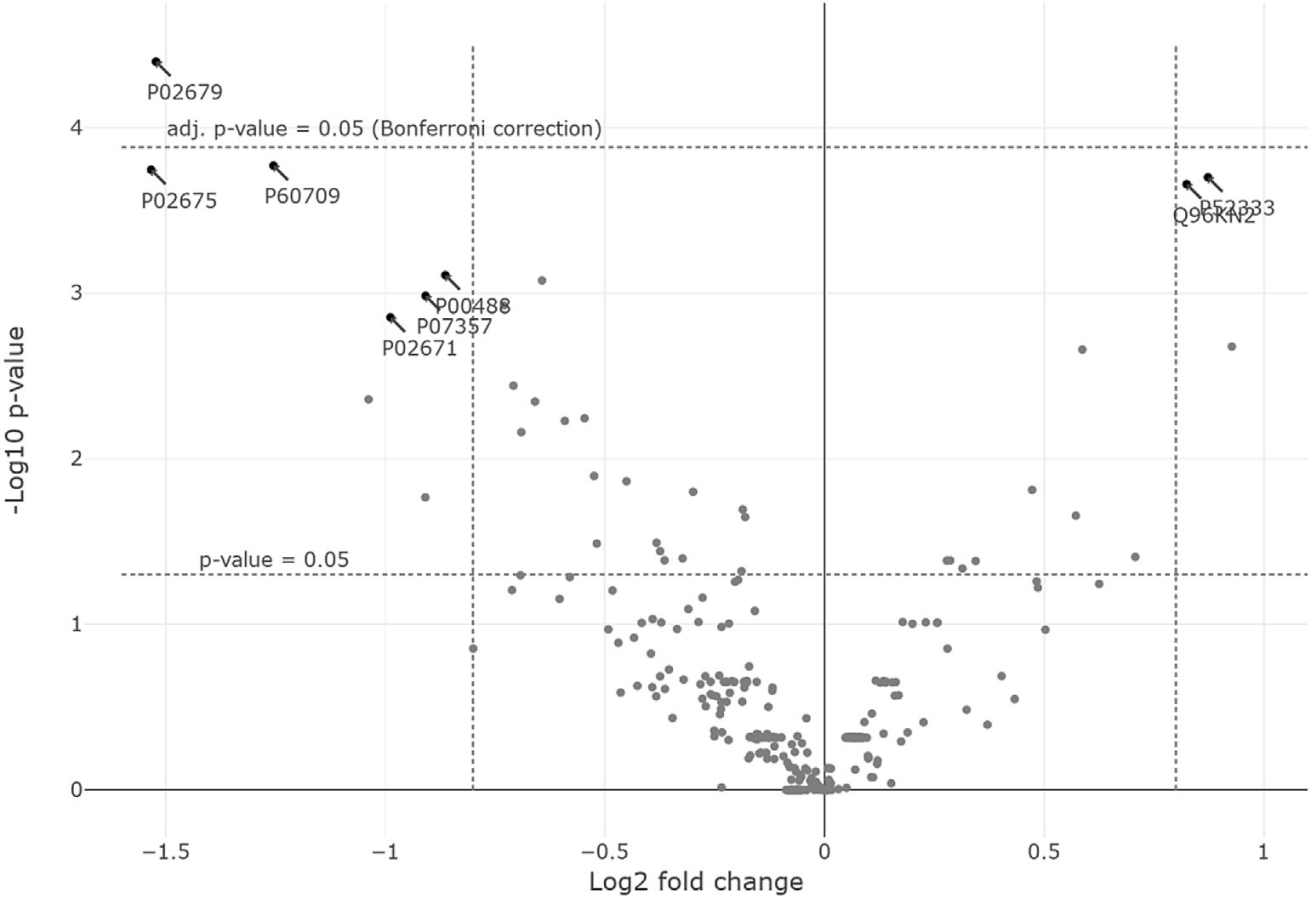
Volcano plot for comparing the relative abundance of proteins (NSAF) between “S” series and “C” series. The log2 expression ratio (biological significance) is plotted versus the –log10 of the p-value obtained from significance testing (permutation analysis). The upper dotted line indicates the threshold for Bonferroni correction. Proteins with UniProt accession number are considered to have significantly changed.

A subsequent comparative analysis of depleted serum samples from children with ASD was performed using these comparison lists. A high (60%) level of matches in the identifications (Fig. 2B) between the control samples and serum samples from children with ASD is observed, which included extensive groups of apolipoproteins (14 proteins), proteins of the blood coagulation system and complement proteins (31 proteins), humoral response proteins (15 proteins), factors of protein activation cascade (26 proteins), and serpins (9 proteins) [1]. In spite of sufficiently high similarity in protein composition of the ASD and control samples, the relative abundance of common proteins in samples is significantly different (Fig. 3).

Fig. 3 also demonstrates the occurrence of proteins quantitatively in the two series of blood serum samples. The higher the occurrence of a certain protein in each sample series, the larger the circle corresponding to this protein is in the Volcano plot (Fig. 3).

We used the Bonferroni correction to adjust for multiple testing and as a guideline for selection of proteins whose content in the two series is reliably different. Six proteins can be isolated near the cut-off threshold for the Bonferroni correction, which are marked with arrows (with corresponding UniProt accession number identifiers displayed) in Fig. 3.

By applying a web-resource DISEASES (RRID:SCR_015664, https://diseases.jensenlab.org/) we found that genes of 5 proteins are associated with cerebrovascular disease and intellectual disability (Table 1). Four proteins – Fibrinogens and Gelsolin mentioned in the reference sources as potential markers are associated with cerebrovascular disease and actin is associated with intellectual disability.

**Table 1:** Group of proteins in samples from “S” series obtained from children with ASD, the content of which differs from the control samples.

№	UniProt AC	Gene's name	Protein's name	Log (Fold change); p-value	DISEASES, Z-score^a^
1	P18206	VCL	Vinculin	−1,72; 0,00038	Vascular disease, 3.3
2	P02679	FGG	Fibrinogen gamma chain	−1,82; 0,00001	Cerebrovascular disease, 2.3
3	P02675	FGB	Fibrinogen beta chain	−1,82; 0,00007	Cerebrovascular disease, 3.7; Atherosclerosis, 2.2
4	P06396	GLS	Gelsolin	−2,0; 0,00002	Neuropathy, 3.8; Polyneuropathy, 3.5
5	P60709	ACTB	Actin, cytoplasmic 1	−2,9; 0,00012	Alzheimer's disease, 3.0; Intellectual disability
6	P02671	FGA	Fibrinogen alpha chain	−2,9; 0,00023	Schizophrenia, 4.9; Cerebrovascular disease, 2.8

a Z-score Є {0; 10}.

**Fig. 4:**
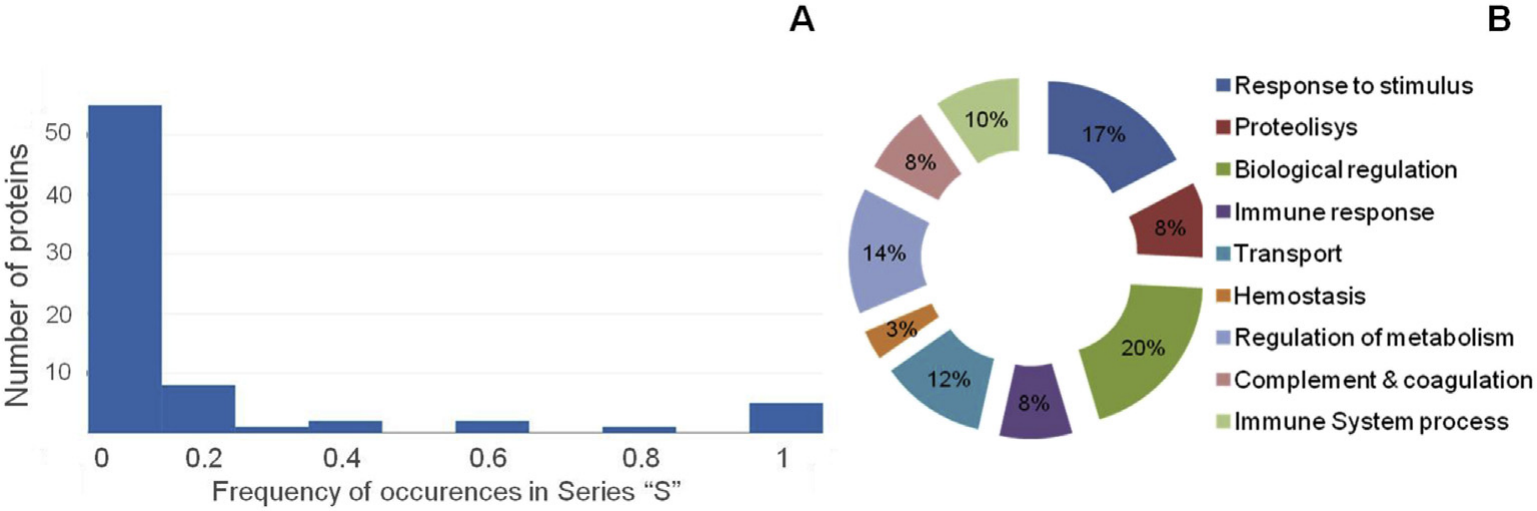
Frequency of occurrence (from 0 to 1) of 6 proteins, the abundance of which in “S” series is lower than in “C” series and 66 proteins specific only for “S” series (А). Analysis of identified proteins clustering by biological functions among the samples of “S” series from children with ASD (g: Profiler) (В).

In addition, we identified 66 proteins that were present only in samples from children with ASD (See Supplementary table 2). We analyzed distribution of biological functions of these proteins using the web-resource Profiler (RRID:SCR_009339, http://biit.cs.ut.ee/gprofiler/), which used Gene Ontology Annotation (GOA). Among the samples in the “S” series we identified functional protein clusters, including proteins taking part in biological regulation (20%), response to stimulus (17%), regulation of metabolism (14%), proteins of immune system process (10%) (Fig. 4).

## 2 Experimental design, materials and methods

### 2.1 Participants

Thirteen children with ASD (mean age of 11 years, IQR = 3) and thirteen healthy control (mean age of 18 years, IQR = 0) children were initially selected. Children in the ASD group were recruited from State educational institution of Krasnodar region « The boarding School № 2», Russia. For the index group, the inclusion criteria consisted of a diagnosis of ASD based on the ICD-10 (International Classification of Diseases, Tenth Revision)/DSM-5 (Diagnostic and Statistical Manual of Mental Disorders, Fives Revision) criteria determined by a child psychiatrist. The ASD diagnosis was further confirmed by the following questionnaires: Autism Diagnostic Interview (ADI-R), Autism Diagnostic Parents Checklist (ADPC) [6]. The patients with severe somatic and neurological pathologies, as well as patients taking long-term pharmacotherapy with an established diagnosis of ASD were excluded. At the time of our research work patients with ASD were treated with behavioral therapy, designed to improve relationships with others and communication. We analyzed samples from children with the following diagnoses (DSM-10) – childhood autism (F84.0, n = 4), atypical autism (F84.1, n = 7) and overactive disorder associated with mental retardation and stereotyped movements (F84.4, n = 2).

The control group consisted of healthy and typically developed children without physical and mental illnesses and was recruited from the same area in order to minimize toxic influences from different environments. Healthy children were also tested by the questionnaires described above. Mental state of children in the control group was within the normal range.

### 2.2 Sample preparation

Serum samples were prepared from venous blood withdrawn from ASD male patients or healthy male volunteers on an empty stomach after an overnight fast.

The samples were provided by the Mental Health Research Center and Laboratory of Molecular Cytogenetics of Neuropsychiatric Diseases, Veltischev Clinical Pediatric Research Institute, Pirogov Russian National Research Medical University (Moscow, Russia). Written informed consents were obtained from the individuals who provided the samples.

The blood was collected in pre-chilled tubes containing ethylenediaminetetraacetic acid (EDTA), quickly mixed, and centrifuged at 4 °C at 1500 revolutions per minute (rpm) for 10 min; and the serum samples were immediately collected and frozen. After centrifugation, the supernatant in the tubes was carefully collected with an automated pipette and placed into 2 mL cryovials [4]. The samples were stored at the temperature of −80 °C, and were not subjected to refreezing. Following enzymatic cleavage, the ASD samples and control samples were mixed. Labeling of biosamples was transparent for two series of comparison.

Forty ml of serum was then brought to a final volume of 160 μl by adding 15 mM MOPS (4-morpholinepropanesulfonic acid sodium salt) solution (рН 7.4). Minor proteins were supplemented using the method of immunoaffinity chromatography on column MARS® Hu-14, 100 mm × 4.6 mm (Agilent) in a gradient consisting of mobile phase A (15mM MOPS, рН 7.4) and mobile phase B (15 mM MOPS, 2 M urea, pH 3.0). For minor protein supplementation, three successive applications of 40 μl of each sample were made. Minor protein fractions were collected within the retention time range from 3.9 to 4.6 min in the isocratic flow of mobile phase A at a flow rate of 0.5 mL/min. The fractions of each sample were combined and dried in vacuum at 30 °C.

The dry residue was reconstituted in 500 μl of 0.1% sodium deoxycholate, 6% acetonitrile and 75 μM triethylammonium bicarbonate (рН 8.5). The protein solution was heated at 90 °C for 10 min under vigorous shaking (1100 rpm). After equilibration to ambient temperature, 3 mM ТСЕР (Tris(2-carboxyethyl)phosphine) was added to the denatured protein solution to restore the sulfhydryl groups of aminoacid residues of cysteine. The reaction was incubated at 45 °C for 20 min. For alkylation, the denatured protein solution was added to the solution of 0.2% 4-vinylpyridine in 30% propan-2-ol up to a final concentration of 0.02% (V/V). The alkylation reaction was carried out for 30 min at a normal temperature in a light proof room.

Enzymatic cleavage of proteins was performed using a specific trypsin protease. The protein solution was added to the modified trypsin at an enzyme-to-substrate ratio of 1:50. After that, the second aliquot of trypsin was added at a ratio of 1:100 and incubation at 37 °C continued for additional 12 h.

After a certain lapse of time, the enzyme reaction was inhibited by adding the formic acid up to the final concentration of 0.5%, which also caused precipitation of insoluble deoxycholic acid. The suspended solids obtained were centrifuged at 12,000 rpm at 15 °C for 10 min. The supernatant (approx. 550 μl) was collected and applied on Discovery DSC solid-phase columns, which were preliminarily equilibrated by a solution of 2% methanol with 0.1% formic acid. After sample application, the columns were washed twice with 1 mL of 0.1% formic acid solution, and then the peptides were eluted from the carrier using the solution of 70% methanol with 5% formic acid in a volume of 1 ml. The eluate collected was dried at 30 °C for 45 min in vacuum. The dry residue was restored in 40 μl of 0.5% formic acid solution and transferred into vials of deactivated glass for mass spectrometric analysis.

### 2.3 Mass spectrometric protein registration

High-performance liquid chromatography-tandem mass spectrometric (HPLC-MS/MS) registration of peptides was carried out using Q Exactive high resolution mass spectrometer (Thermo Scientific, USA, Catalog # IQLAAEGAAPFALGMBDK) by chromatographic separation using Ultimate 3000 Nano-flow HPLC system (Thermo Scientific, USA, Catalog # ULTIM3000RSLCNANO). Peptides in a volume of 5 μl were applied on an enrichment column PepMap C18 for 4 min in the isocratic flow of mobile phase C (2% acetonitrile, 0.08% formic acid, 0.015% trifluoroacetic acid) at a flow rate of 20 μl/min. Peptides were separated using Acclaim PepMap C18 analytical column in the nano-flow mode in a linear gradient of mobile phase A (0.08% formic acid, 0.015% trifluoroacetic acid) and mobile phase B (0.08% formic acid, 0.015% trifluoroacetic acid in acetonitrile) at a flow rate of 400 nl/min; the initial ratio А:В was 98:2. Separation was performed using a gradient elution from 2% to 35% of mobile phase B for 80 min, followed by column washing at 90% of phase B for 10 min with subsequent system equilibration at initial gradient conditions for 20 min [5].

Registration of peptide signal was carried out in the dependent tandem scan mode. After prescanning of precursory ions with maximum accumulation time not more than 80 ms with resolution R = 70 K in the range of 420–1250 *m*/*z*, ions with charge state z = 2 + to 5 + were selected for tandem scanning using dynamic exclusion for the duration of one half -width of the chromatographic peak (but not for more than 15 s). Isolation of precursory ions was performed with the width of w = ± 1 Th within the range from 9 to 17 s from the peak apex for the tandem scanning. HPLC-MS/MS spectra in the RAW format were processed in Mass Hunter version В 2.0 [7].

### 2.4 Protein identification

The peak lists obtained from the MS/MS spectra were identified using OMSSA version 2.1.9 [8]. The search was conducted using SearchGUI version 3.2.20 (RRID:SCR_012054) [9]. Protein identification was conducted against a concatenated target/decoy [10] version of the Homo sapiens complement of the UniProtKB 88,703 target sequences (RRID:SCR_004426, https://www.uniprot.org/) [11]. Decoy sequences were created by reversing the target sequences in SearchGUI. The identification settings were as follows: Trypsin specificity was applied with a maximum of two missed cleavages: 10.0 ppm as MS1 and 0.05 Da as MS2 tolerances; variable modifications: oxidation of M (+15.994,915 Da), deamidation of Q (+0.984,016 Da), and carbamidomethylation of C (+57.021,464 Da). Peptide spectrum matches (PSMs), peptides, and proteins were validated at a 1.0% false discovery rate (FDR) and estimated using the decoy hit distribution. Spectrum counting abundance indexes were estimated using the normalized spectrum relative abundance factor (NSAF) adapted for better handling of protein inference issues and peptide detectability [12]. We have revealed 2 outliers in the control series, which were excluded from the analysis.

### 2.5 Permutation analysis

A permutation analysis with 10^5^ permutations was used to determine the statistical significance of the NSAF differences between “S” series and “C” series. As statistics, the difference in means values of ln(NSAF) for each protein T = ln(NSAF_“S”_) – ln(NSAF_“C”_) was used. As a null hypothesis, this difference was taken to be equal to zero (*H*_*0*_*: T* = *0*). After determination of all protein identifications in each series, we performed a permutation analysis on those protein identifications that are found in both series. If there was no NSAF value, it was taken to be equal to 1e-6. Since the distribution of NSAF values and the corresponding logarithms is unknown, and also due to the small sample size, a permutation analysis was applied. Advantages of permutation testing are the independent of distribution form, the suitability for small sample size groups, and the possibility of using any statistics of interest.

To calculate p-values obtained in permutation test were adjusted with Bonferroni correction. The number of permuted *T* values (*T*_*i*_) greater or equal to the original *T* value divided to number of permutations (*N*):

$$p=\frac{1}{N}\overset{N}{\underset{i=1}{\sum }}I\left(\left|T_{i}\right|\geq \left|T\right|\right).$$

Note, *P*-values, which appeared to be zero after the calculation using this equation, were taken to be equal to 1/*N*.
